# Development of 1,2,4-Triazole-5-Thione Derivatives as Potential Inhibitors of Enoyl Acyl Carrier Protein Reductase (InhA) in Tuberculosis.

**DOI:** 10.22037/ijpr.2019.112039.13495

**Published:** 2019

**Authors:** Dhagash Vora, Neha Upadhyay, Kalpana Tilekar, Viral Jain, C S Ramaa

**Affiliations:** *Department of Pharmaceutical Chemistry, Bharati Vidyapeeth’s College of Pharmacy, Navi Mumbai-400614, Maharashtra, India.*

**Keywords:** Mycobacterium tuberculosis, 1, 2, 4-Triazole-5-thiones, InhA inhibition, ADME, REMA.

## Abstract

Tuberculosis (TB) ranks second, next to AIDS making it most formidable disease in the present age. One of the crucial enzymes involved in cell wall synthesis of *Mycobacterium tuberculosis*, InhA (enoyl acyl carrier protein reductase), one of the crucial enzymes involved in cell wall synthesis of *Mycobacterium tuberculosis*, has been authenticated as an effective target for anti-mycobacterial drug development. In the current work, novel derivatives of 1,2,4-triazole-5-thione rationally designed, synthesized and spectrally characterized as promising InhA inhibitors. Anti-mycobacterial potential was determined by resazurin microtiter assay using Mtb H_37_Rv strain. The mechanism of action of these compounds was confirmed by InhA enzyme inhibition studies. 6b, the most active compound of the series displayed MIC of 0.19 µM in resazurin microtiter assay and InhA inhibition with IC_50_ of 90 nM.

## Introduction

Amongst the most formidable diseases of the present age, tuberculosis (TB) ranks second, next to AIDS, and it needs to be looked upon with utmost priority. The World Health Organization’s (WHO) 2018 global report identified *Mycobacterium tuberculosis* (Mtb) infection as one of the major causes of global mortality and morbidity and the resulting disease, TB, caused an estimated 1.6 million deaths out of 11.1 million people who fell ill with it in 2017 (1). In 2018, major cases of new tuberculosis arose in Asia, which contributed 60% of new cases globally. Looking at India, around 100 people die every day due to TB (2, 3). This situation has further worsen owing to an increase in multidrug resistant tuberculosis and extensively drug resistant tuberculosis (MDRTB and XDRTB respectively) cases in the past decade. Adding to the more fatal aspect of TB, recently, cases of totally drug-resistant tuberculosis (TDR-TB) have been identified wherein the patients do not respond to any of the available anti-TB drugs thereby making TB the nastiest and most noxious diseases than ever before (4).

Mtb comprises unique fatty acids, the mycolic acids, which are oddly lengthy chain β-hydroxy fatty acids with a long α-alkyl side chain (5). These are the chief building blocks of the protecting layer in the cell envelope of Mtb. Mycolic acids in Mtb are formed by a saturated short fatty acyl chain of 20-26 carbon atoms and a long meromycolic acid chain of 50-60 carbon atoms. The biosynthesis of mycolic acids is achieved by the fatty acid synthase system (FAS) in *M. tuberculosis. *Mtb has unique FAS-I and FAS-II fatty acids biosynthetic pathways which are distinct from other bacteria. Amongst the various enzymes involved in FAS-II pathway, the NADH-dependent *trans*-2-enoyl acyl carrier protein reductase (InhA) is the main catalyst in biosynthesis of mycolic acid. Many literature reports have highlighted InhA as a prime molecular target of isoniazid (1), which is used as a first-line agent in the treatment of TB. INH is a prodrug, which is activated by KatG (a catalase-peroxidase) by oxidation to an acyl radical which binds covalently to co-substrate, NAD^+^. The INH-NAD^+ ^complex then acts as an effective InhA inhibitor (6-8). 

**Figure 1:**
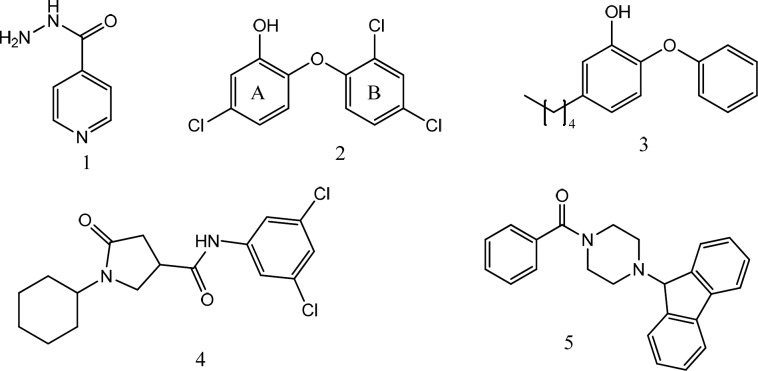
InhA inhibitors from literature; Isoniazid (1), Triclosan (2), Diphenyl ether derivative (3), Pyrrolidine carboxamide derivative (4) and Piperazine derivative (5)

The necessity for INH activation unlocked a way-in for the development of Mtb drug resistance. Thus, direct inhibition of InhA, will bypass the compulsory activation step of INH, and will serve as a promising target in the development of novel agents in anti-tubercular therapy. Other than triclosan (**2**) (9), which is a nonselective and relatively weak agent, three series of direct InhA inhibitors, namely, diphenyl ether derivatives (**3**) (10-12), pyrrolidine carboxamide derivatives (**4**), and piperazine derivatives (arylamides) (**5**) (Figure 1), have exhibited potent *in-vitro* activity. In this work, we report our findings of 1,2,4-triazole-5-thiones and 1,3,4-oxadiazole-2-thiones as a novel series of InhA inhibitors.

## Experimental

*Chemistry*

*General*

**Scheme 1:**
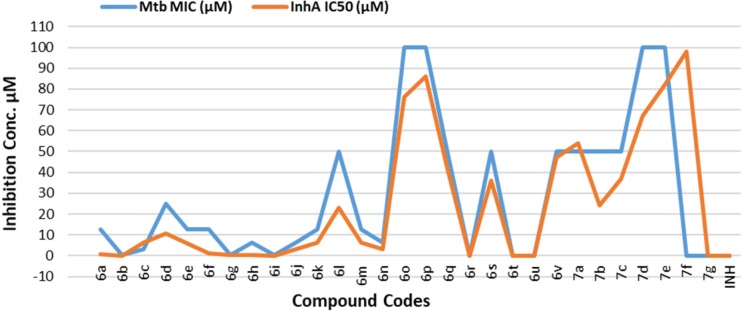
Experimental scheme for synthesis of 1,2,4-triazole-5-thione derivatives and 1,3,4-oxadiazole-2-thione derivatives; i. CS_2_, Δ, EtOH, KOH, ii. NH_2_-NH_2_.H_2_O, Δ, Glacial acetic acid, iii. K_2_CO_3_, DCM, iv. K_2_CO_3_, DMF, RT, v. K_2_CO_3_, DMF, RT

Chemicals and solvents of LR grade were used for synthesis and purchased from Research Lab, S D Fine and Sigma suppliers in India. The reactions were monitored using pre-coated TLC plates (Merck pre-coated Silica Gel 60 F_254_) using various solvent systems. Veego melting point apparatus was used for recording of Melting points which were uncorrected. The synthesized compounds were structurally characterized using FTIR, NMR. Infrared spectroscopy was carried on the Shimadzu FT/IR-8400S. ^1^HNMR spectra were determined by Varian (300 MHz and 600MHz) and Bruker (400 MHz and 500MHz) NMR spectroscopies, whereas ^13^C-NMR were recorded on Varian (75 MHz) and Bruker (100 MHz and 125 MHz) NMR spectroscopies. Chemical shifts values are described in ppm (δ) against TMS as internal standard. The designations for signals are as follows: s-singlet; d-doublet; dd-doublet of doublet; t-triplet; and m-multiplet. General synthetic scheme (Scheme 1) was followed for the synthesis of 1,2,4-triazole-5-thione and 1,3,4-oxadiazole-2-thione derivatives.

*Synthesis of 5-pyridin-4-yl-3H-(1,3,4)-oxadiazole-2-thione* ***(4)***

In ethanolic solution of KOH (0.1 mol), isonicotinic acid hydrazide **3** (0.1 mol) was dissolved and to this carbon disulfide (0.1 mol) was added drop wise. The reaction was then refluxed for 10-12 h. After completion the mixture was poured over crushed ice and acidified with conc. HCl. The precipitate was filtered and recrystallized by using ethanol (13, 14).

Yellow crystalline solid; Yield: 71%; M.p.: 263-265 °C; FT-IR (KBr, cm^-1^): 3325 (N-H stretch, oxadiazole), 2968, 2933 (aromatic C-H stretch), 1637 (C=N stretch, oxadiazole), 1612, 1560 (aromatic C=C stretch), 1165 (C=S stretch, oxadiazole), 1016 (C-O-C stretch, oxadiazole); ^1^H NMR (400 MHz, DMSO-d_6_): δ 8.82 (d, *J *= 4.0 Hz, 2H, pyridine), 7.82 (d, *J *= 4.0Hz, 2H, pyridine) ^13^C NMR (100 MHz, DMSO-d_6_): δ 177.05 (1C, C-2 [C=S], oxadiazole), 158.78 (1C, C-5, oxadiazole), 150.85, 129.70, 119.62 (5C, pyridine).

*Synthesis of 4-amino-3-(pyridin-4-yl)-1H-(1,2,4)-triazole-5-thione (* ***5*** *)*

5-pyridin-4-yl-3H-(1,3,4)-oxadiazole-2-thione (**4**) (0.05 mol) was refluxed with hydrazine hydrate (99%, 75 mL) for 4 h. The cooled reaction mixture was quenched with ice-cold water followed by acidification with glacial acetic acid, to get crude which was purified by recrystallization from ethanol [15].

Pale yellow crystalline solid; Yield: 68%; M.p.: 256-258 °C; FT-IR (KBr, cm^-1^): 3271, 3163 (N-H stretch, Amine [NH_2_]), 3234 (N-H stretch, triazole), 3090, 3057 (aromatic C-H stretch), 1606 (C=N stretch, triazole), 1572, 1556 (aromatic C=C stretch), 1217 (C=S stretch, triazole); ^1^H NMR (500 MHz, DMSO-d_6_): δ 8.77 (dd, *J *= 5.0, 1.5 Hz, 2H, pyridine), 8.03 (dd, *J *= 4.5, 1.5 Hz, 2H, pyridine), 5.86 (s, 2H, NH_2_) ^13^C NMR (125 MHz, DMSO-d_6_): δ 168.15 (1C, C-5 [C=S], triazole), 147.82 (1C, C-3, triazole), 150.62, 133.36, 122.01 (5C, pyridine).

*General synthetic procedure for the preparation of 2-chloro-N-(aryl or heteroaryl) acetamides (* ***2a-2y*** *)*

We have previously published synthetic procedure and characterization of 2a-2y in our different reports (16-19).

*General synthetic procedure for the preparation of* ***6a-6v***

A mixture of 4-amino-3-(pyridin-4-yl)-1H-(1,2,4)-triazole-5-thione (**5**) (0.05 mol) and 2-chloro-N-(aryl or heteroaryl) acetamides (**2a-2v**) (0.05 mol) along with anhydrous potassium carbonate (K_2_CO_3_) (0.075 mol) was stirred in dimethyl formamide (DMF) at room temperature. After completion of reaction (monitored by TLC), ice cold water was added to precipitate solid which was filtered and purified by recrystallization in appropriate solvent.

*2-(4-Amino-3-pyridin-4-yl-5-thioxo-4,5-dihydro-[1,2,4]triazol-1-yl)-N-phenyl acetamide (* ***6a*** *)*

White amorphous solid; Recrystallizing solvent: Ethanol; Yield: 69%; M.p.: 242-244 °C; FT-IR (KBr, cm^-1^): 3234, 3192 (N-H stretch, Amine [NH_2_]), 3294 (N-H stretch, amide), 3051, 3039 (aromatic C-H stretch), 2976, 2875 (aliphatic C-H stretch, CH_2_), 1660 (C=O stretch, amide), 1188 (C=S stretch, triazole); ^1^H NMR (400 MHz, DMSO-d_6_): δ10.34 (s, 1H, NH),8.71 (d, *J *= 3.2 Hz, 2H, pyridine), 7.97 (d, *J *= 3.2 Hz, 2H, pyridine), 7.56 (d, *J *= 5.2 Hz, 2H, phenyl), 7.29 (t, *J *= 5.0 Hz, 2H, phenyl), 7.04 (t, *J *= 4.0 Hz, 1H, phenyl), 6.31 (s,2H, NH_2_), 4.17 (s, 2H, CH_2_);^13^C NMR (100 MHz, DMSO-d_6_): δ 165.88 (1C, C-5 [C=S], triazole), 154.69 (1C, C=O, amide), 147.95 (1C, C-3, triazole), 151.94, 133.87, 121.24 (5C, pyridine), 138.73, 128.65, 123.43, 119.11 (6C, phenyl), 36.29 (1C, CH_2_). Anal. Calc. for C_15_H_14_N_6_OS: C, 55.20; H, 4.32; N, 25.75.

*2-(4-amino-3-(pyridin-4-yl)-5-thioxo-4,5-dihydro-1H-1,2,4-triazol-1-yl)-N-(p-tolyl)acetamide (* ***6b*** *)*

White amorphous solid; Recrystallizing solvent: Ethanol; Yield: 84%; M.p: 237-238 °C; FT-IR (KBr, cm^-1^): 3234, 3176 (N-H stretch, Amine [NH_2_]), 3296 (N-H stretch, amide), 3051, 3032 (aromatic C-H stretch), 2974, 2920, 2874 (aliphatic C-H stretch, CH_3_ and CH_2_), 1664 (C=O stretch, amide), 1203 (C=S stretch, triazole); ^1^H NMR (500 MHz, DMSO-d_6_): δ 10.27 (s, 1H, NH), 8.74 (dd, *J *= 4.5, 1.5 Hz, 2H, pyridine), 8.00 (dd, *J *= 4.5, 1.5 Hz, 2H, pyridine), 7.47 (d, *J *= 8.5 Hz, 2H, phenyl), 7.12 (d, *J *= 8.0Hz, 2H, phenyl), 6.33 (s,2H, NH_2_), 4.18 (s, 2H, CH_2_), 2.25 (s, 3H, CH_3_); ^13^C NMR (125 MHz, DMSO-d_6_): δ 166.03 (1C, C-5 [C=S], triazole), 155.21 (1C, C=O, amide), 150.54 (1C, C-3, triazole), 152.43, 134.32, 121.73 (5C, pyridine), 136.74, 132.90, 129.61, 119.57 (6C, phenyl), 36.61 (1C, CH_2_), 20.87 (1C, CH_3_). Anal.Calc. for C_16_H_16_N_6_OS: C, 56.45; H, 4.74; N, 24.69.

*2-(4-amino-3-(pyridin-4-yl)-5-thioxo-4,5-dihydro-1H-1,2,4-triazol-1-yl)-N-(4-bromo-2-fluorophenyl)acetamide (* ***6c*** *)*

White amorphous solid; Recrystallizing solvent: Isopropanol; Yield: 74%; M.p: 276-278 °C; FT-IR (KBr, cm^-1^): 3246, 3182 (N-H stretch, Amine [NH_2_]), 3340 (N-H stretch, amide), 3043, 3009 (aromatic C-H stretch), 2931 (aliphatic C-H stretch, CH_2_), 1674 (C=O stretch, amide), 1336 (C-F stretch), 1176 (C=S stretch, triazole); ^1^H NMR (600 MHz, DMSO-d_6_): δ 10.25 (s, 1H, NH), 8.71 (d, *J *= 5.4 Hz, 2H, pyridine), 7.98 (d, *J *= 5.4 Hz, 2H, pyridine), 7.90 (t, *J *= 8.7 Hz, 1H, phenyl), 7.60 (d, *J *= 10.8 Hz, 1H, phenyl), 7.37 (d, *J *= 9.0Hz, 1H, phenyl), 6.30 (s, 2H, NH_2_), 4.16 (s, 2H, CH_2_); ^13^C NMR (100 MHz, DMSO-d_6_): δ 166.59 (1C, C-5 [C=S], triazole), 154.43 (1C, C=O, amide), 151.89 (1C, C-3, triazole), 149.66, 133.86, 121.21 (5C, pyridine), 153.93, 127.88, 125.56, 124.83, 118.58, 115.54 (6C, phenyl), 36.13 (1C, CH_2_). Anal.Calc. for C_15_H_12_BrFN_6_OS: C, 42.56; H, 2.86; N, 19.86.

*2-(4-Amino-3-(pyridin-4-yl)-5-thioxo-4,5-dihydro-1H[1,2,4]triazol-1-yl)-N-(4-methoxyphenyl) acetamide (* ***6d*** *)*

Dark brown amorphous solid; Recrystallizing solvent: Ethanol; Yield: 79%; M.p.: 256-258^o^C; FT-IR (KBr, cm^-1^): 3234, 3174 (N-H stretch, Amine [NH_2_]), 3290 (N-H stretch, amide), 3132, 3041 (aromatic C-H stretch), 2962, 2920, 2881 (aliphatic C-H stretch, CH_3_ and CH_2_), 1658 (C=O stretch, amide), 1247 (C-O-C stretch), 1188 (C=S stretch, triazole); ^1^H NMR (500 MHz, DMSO-d_6_): δ 10.22 (s, 1H, NH), 8.74 (d, *J *=6.0 Hz, 2H, pyridine), 8.01 (d, *J *= 5.5 Hz, 2H, pyridine), 7.49 (d, *J *= 9.0Hz, 2H, phenyl), 6.89 (d, *J *=9.0 Hz, 2H, phenyl), 6.33 (s,2H, NH_2_), 4.16 (s, 2H, CH_2_), 3.33 (s, 3H, CH_3_); ^13^C NMR (125 MHz, DMSO-d_6_): δ 165.76 (1C, C-5 [C=S], triazole), 155.20 (1C, C=O, amide), 152.42 (1C, C-3, triazole), 150.34, 134.33, 121.73 (5C, pyridine), 155.82, 132.37, 121.12, 114.35 (6C, phenyl), 55.59 (1C, CH_3_), 36.55 (1C, CH_2_). Anal. Calc. for C_16_H_16_N_6_O_2_S: C, 53.92; H, 4.52; N, 23.58.

*2-(4-Amino-3-(pyridin-4-yl)-5-thioxo-4,5-dihydro-1H[1,2,4]triazol-1-yl)-N-(3-trifluoromethylphenyl) acetamide (6* ***e*** *)*

White amorphous solid; Recrystallizing solvent: Ethanol; Yield: 54%; M.p.: 242-244 °C; FT-IR (KBr, cm^-1^): 3211, 3190 (N-H stretch, Amine [NH_2_]), 3298 (N-H stretch, amide), 3074, 3026 (aromatic C-H stretch), 2993, 2953 (aliphatic C-H stretch, CH_2_), 1689 (C=O stretch, amide), 1323 (C-F stretch), 1180 (C=S stretch, triazole); ^1^H NMR (600 MHz, DMSO-d_6_): δ 10.73 (s, 1H, NH), 8.74 (d, *J *= 5.4 Hz, 2H, pyridine), 7.99 (d, *J *= 5.4 Hz, 2H, pyridine), 8.09, (s, 1H, phenyl), 7.77 (d, *J *= 7.8 Hz, 1H, phenyl), 7.58 (t, *J *= 8.1 Hz, 1H, phenyl), 7.44 (d, *J *= 8.4 Hz, 1H, phenyl), 6.35 (s,2H, NH_2_), 4.23 (s, 2H, CH_2_); ^13^C NMR (100 MHz, DMSO-d_6_): δ 166.39 (1C, C-5 [C=S], triazole), 154.58 (1C, C=O, amide), 149.89 (1C, C-3, triazole), 151.96, 133.85, 122.51 (5C, pyridine), 139.46, 129.70, 125.24, 121.21, 119.68, 115.27 (6C, phenyl), 129.84 (1C, CF_3_), 36.15 (1C, CH_2_). Anal. Calc. for C_16_H_13_F_3_N_6_OS: C, 48.73; H, 3.32; N, 21.31.

*2-(4-Amino-(3-pyridin-4-yl)-5-thioxo-4,5-dihydro-1H[1,2,4]triazol-1-yl)-N-(4-chlorophenyl) acetamide (6* ***f*** *)*

White amorphous solid; Recrystallizing solvent: Ethanol; Yield: 66%; M.p.: 218-220 °C; FT-IR (KBr, cm^-1^): 3284, 3223 (N-H stretch, Amine [NH_2_]), 3346 (N-H stretch, amide), 3115, 3037 (aromatic C-H stretch), 2918, 2872 (aliphatic C-H stretch, CH_2_), 1662 (C=O stretch, amide), 1180 (C=S stretch, triazole), 742 (C-Cl stretch); ^1^H NMR (500 MHz, DMSO-d_6_): δ10.51 (s, 1H, NH), 8.74 (d, *J *= 6.0Hz, 2H, pyridine), 8.00 (d, *J *= 6.0 Hz, 2H, pyridine), 7.62 (d, *J *= 8.5 Hz, 2H, phenyl), 7.38 (d, *J *= 9.0 Hz, 2H, phenyl), 6.33 (s,2H, NH_2_), 4.20 (s, 2H, CH_2_); ^13^C NMR (125 MHz, DMSO-d_6_): δ 166.49 (1C, C-5 [C=S], triazole), 155.18 (1C, C=O, amide), 150.54 (1C, C-3, triazole), 152.46, 134.29, 121.72 (5C, pyridine), 138.20, 129.17, 127.49, 121.08 (6C, phenyl), 36.15 (1C, CH_2_). Anal. Calc. for C_15_H_13_ClN_6_OS: C, 49.93; H, 3.63; N, 23.29.

*2-(4-Amino-(3-pyridin-4-yl)-5-thioxo-4,5-dihydro-1H[1,2,4]triazol-1-yl)-N-(3,4-dimethylphenyl) acetamide (* ***6g*** *)*

Pale brown amorphous solid; Recrystallizing solvent: Ethanol; Yield: 56%; M.p.: 242-244 °C; FT-IR (KBr, cm^-1^): 3234, 3180 (N-H stretch, Amine [NH_2_]), 3292 (N-H stretch, amide), 3036 (aromatic C-H stretch), 2976, 2947, 2920, 2870 (aliphatic C-H stretch, CH_3_ and CH_2_), 1662 (C=O stretch, amide), 1213 (C=S stretch, triazole); ^1^H NMR (600 MHz, DMSO-d_6_): δ 10.17 (s, 1H, NH), 8.71 (d, *J *= 4.8 Hz, 2H, pyridine), 7.98 (d, *J *= 5.4 Hz, 2H, pyridine), 7.33 (s, 1H, phenyl), 7.27 (d, *J *= 8.4 Hz, 1H, phenyl), 7.04 (d, *J *= 8.4 Hz, 1H, phenyl), 6.30 (s,2H, NH_2_), 4.14 (s, 2H, CH_2_), 2.16, 2.14 (s, 6H, 2 CH_3_); ^13^C NMR (100 MHz, DMSO-d_6_): δ 165.44 (1C, C-5 [C=S], triazole), 154.54 (1C, C=O, amide), 149.64 (1C, C-3, triazole), 151.80, 134.03, 121.24 (5C, pyridine), 136.26, 136.17, 131.22, 129.39, 120.39, 116.71 (6C, phenyl), 36.58 (1C, CH_2_), 18.76, 19.56 (2C, 2 CH_3_). Anal. Calc. for C_17_H_18_N_6_OS: C, 57.61; H, 5.12; N, 23.71.

*2-(4-Amino-(3-pyridin-4-yl)-5-thioxo-4,5-dihydro-1H[1,2,4]triazol-1-yl)-N-(3-chloro-4-methylphenyl) acetamide (6* ***h*** *)*

Buff solid; Recrystallizing solvent: Ethanol; Yield: 85%; M.p: 214-216 °C; FT-IR (KBr, cm^-1^): 3263, 3182 (N-H stretch, Amine [NH_2_]), 3298 (N-H stretch, amide), 3099, 3037 (aromatic C-H stretch), 2958, 2922, 2864 (aliphatic C-H stretch, CH_3_ and CH_2_), 1662 (C=O stretch, amide), 1186 (C=S stretch, triazole), 775 (C-Cl stretch); ^1^H NMR (500 MHz, DMSO-d_6_): δ 10.47 (s, 1H, NH), 8.74 (d, *J *= 5.5 Hz, 2H, pyridine), 8.00 (d, *J *= 5.5 Hz, 2H, pyridine), 7.79 (s, 1H, phenyl), 7.35 (d, *J *= 8.5 Hz, 1H, phenyl), 7.29 (d, *J *= 8.5 Hz, 1H, phenyl), 6.33 (s,2H, NH_2_), 4.19 (s, 2H, CH_2_), 2.28 (s, 3H, CH_3_); ^13^C NMR (100 MHz, DMSO-d_6_): δ 166.48 (1C, C-5 [C=S], triazole), 155.15 (1C, C=O, amide), 150.54 (1C, C-3, triazole), 152.15, 134.30, 121.73 (5C, pyridine), 138.30, 133.47, 131.70, 130.60, 119.50, 118.19 (6C, phenyl), 36.46 (1C, CH_2_), 19.36 (1C, CH_3_). Anal. Calc. for C_16_H_15_ClN_6_OS: C, 51.27; H, 4.03; N, 22.42.

*2-(4-Amino-(3-pyridin-4-yl)-5-thioxo-4,5-dihydro-1H[1,2,4]triazol-1-yl)-N-(3-methylphenyl)acetamide (6* ***i*** *)*

Gray amorphous solid; Recrystallizing solvent: Ethanol; Yield: 74%; M.p.: 225-227 °C; FT-IR (KBr, cm^-1^): 3234, 3176 (N-H stretch, Amine [NH_2_]), 3298 (N-H stretch, amide), 3053, 3031 (aromatic C-H stretch), 2974, 2920, 2874 (aliphatic C-H stretch, CH_3_ and CH_2_), 1664 (C=O stretch, amide), 1205 (C=S stretch, triazole); ^1^H NMR (500 MHz, DMSO-d_6_): δ 10.28 (s, 1H, NH), 8.74 (d, *J *= 6.0Hz, 2H, pyridine), 8.00 (d, *J *= 6.0Hz, 2H, pyridine), 7.42 (s, 1H, phenyl), 7.36 (d, *J *= 8.0Hz, 1H, phenyl), 7.20 (t, *J *= 7.75 Hz, 1H, phenyl),6.89 (d, *J *= 7.5 Hz, 1H, phenyl), 6.33 (s,2H, NH_2_), 4.18 (s, 2H, CH_2_), 2.28 (s, 3H, CH_3_); ^13^C NMR (125 MHz, DMSO-d_6_): δ 166.22 (1C, C-5 [C=S], triazole), 155.21 (1C, C=O, amide), 150.54 (1C, C-3, triazole), 152.43, 134.32, 121.73 (5C, pyridine), 139.16, 138.44, 129.07, 124.66, 120.08, 116.78 (6C, phenyl), 36.63 (1C, CH_2_), 21.60 (1C, CH_3_). Anal. Calc. for C_16_H_16_N_6_OS: C, 56.45; H, 4.74; N, 24.69.

*2-(4-Amino-(3-pyridin-4-yl)-5-thioxo-4,5-dihydro-1H[1,2,4]triazol-1-yl)-N-(3-chlorophenyl) acetamide (6* ***j*** *)*

White crystalline solid; Recrystallizing solvent: Ethanol; Yield: 65%; M.p.: 220-222 °C; FT-IR (KBr, cm^-1^): 3257, 3227 (N-H stretch, Amine [NH_2_]), 3336 (N-H stretch, amide), 3103, 3064 (aromatic C-H stretch), 2926, 2862 (aliphatic C-H stretch, CH_2_), 1670 (C=O stretch, amide), 1178 (C=S stretch, triazole), 785 (C-Cl stretch); ^1^H NMR (600 MHz, DMSO-d_6_): δ 10.54 (s, 1H, NH), 8.71 (d, *J *= 5.4 Hz, 2H, pyridine), 7.97 (d, *J *= 5.4 Hz, 2H, pyridine), 7.78 (s, 1H, phenyl), 7.41 (d, *J *= 7.8 Hz, 1H, phenyl), 7.33 (t, *J *= 8.1 Hz, 1H, phenyl),7.11 (d, *J *= 7.8 Hz, 1H, phenyl), 6.31 (s,2H, NH_2_), 4.18 (s, 2H, CH_2_); ^13^C NMR (100 MHz, DMSO-d_6_): δ 166.13 (1C, C-5 [C=S], triazole), 154..55 (1C, C=O, amide), 149.74 (1C, C-3, triazole), 151.89, 133.94, 121.22 (5C, pyridine), 140.04, 133.25, 130.02, 123.09, 118.72, 117.35 (6C, phenyl), 36.30 (1C, CH_2_). Anal. Calc. for C_15_H_13_ClN_6_OS: C, 49.93; H, 3.63; N, 23.29.

**Figure 2:**
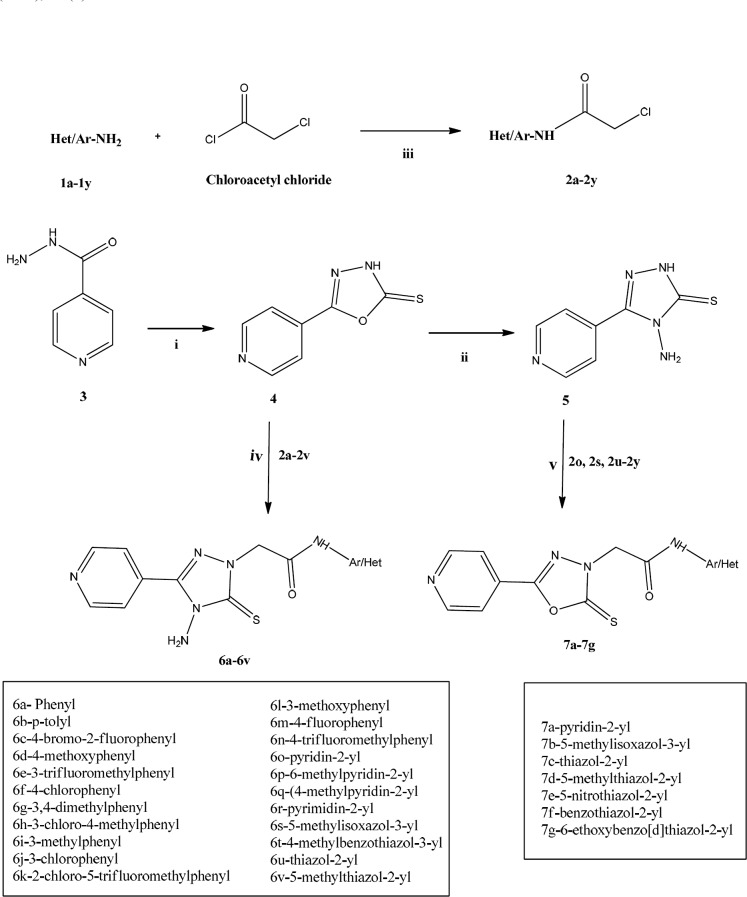
Designing strategy adopted for 1,2,4-triazole-5-thione compounds; The six atoms chain length between the rings A and C has been shown by *.

**Figure 3.**
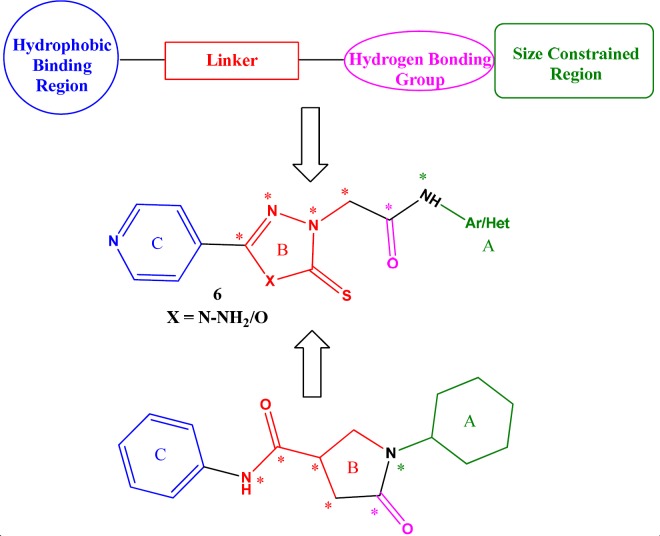
Spread of compounds on Boiled Egg Plot. The white region is for high probability of passive absorption by the gastrointestinal tract, and the yellow region (yolk) is for high probability of brain penetration. Yolk and white areas are not mutually exclusive. In addition, the points are coloured in blue if predicted as actively effluxed by P-gp (PGP+) and in red if predicted as non-substrate of P-gp (PGP−)

**Figure 4:**
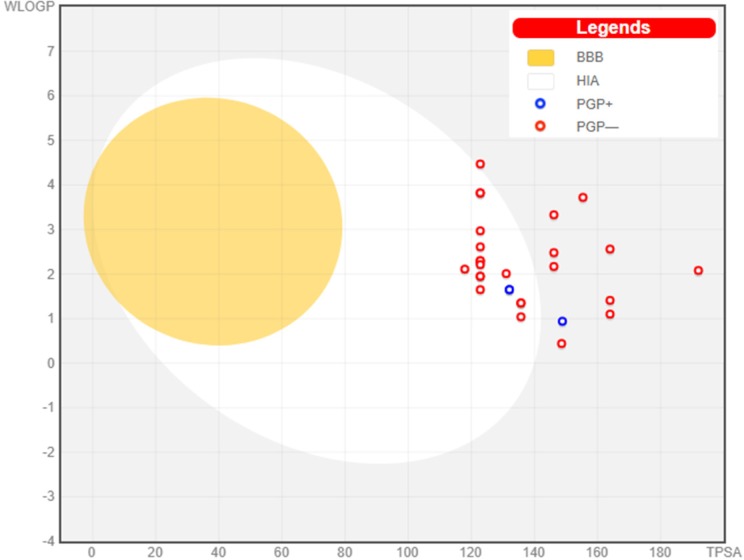
Graphical representation of correlation between Mtb MIC and InhA IC50

**Table 1:** SwissADME prediction data

**Code**	**TPSA** **a**	**Log P** **b**	**Log S** **c**	**ESOL** **d ** **Class**	**GI** **e ** **absorption**	**Lipinski #violations**	**Lead likeness #violations**
6a	122.85	1	-2.65	Soluble	High	0	0
6b	122.85	1.66	-2.94	Soluble	High	0	0
6c	122.85	1.76	-3.71	Soluble	High	0	1
6d	132.08	1.14	-2.71	Soluble	High	0	1
6e	122.85	1.88	-3.48	Soluble	High	0	1
6f	122.85	1.52	-3.24	Soluble	High	0	1
6g	122.85	1.9	-3.24	Soluble	High	0	1
6h	122.85	2.17	-3.53	Soluble	High	0	1
6i	122.85	1.66	-2.94	Soluble	High	0	0
6j	122.85	1.52	-3.24	Soluble	High	0	1
6k	122.85	2.12	-4.08	Moderately soluble	Low	0	1
6l	132.08	1.14	-2.71	Soluble	High	0	1
6m	122.85	1.39	-2.81	Soluble	High	0	0
6n	122.85	1.88	-3.48	Soluble	High	0	1
6o	135.74	0.36	-2.19	Soluble	High	0	0
6p	135.74	1.02	-2.51	Soluble	High	0	0
6q	135.74	1.02	-2.48	Soluble	High	0	0
6r	148.63	-0.27	-1.79	Very soluble	Low	0	0
6s	148.88	0.17	-2.14	Soluble	Low	0	0
6t	163.98	1.64	-3.82	Soluble	Low	0	1
6u	163.98	-0.14	-2.29	Soluble	Low	0	0
6v	163.98	0.54	-2.6	Soluble	Low	0	0
7a	117.93	0.48	-2.85	Soluble	High	0	0
7b	131.07	-0.13	-2.79	Soluble	High	0	0
7c	146.17	-0.02	-2.94	Soluble	Low	0	0
7d	146.17	0.25	-3.26	Soluble	Low	0	0
7e	191.99	-0.06	-3.19	Soluble	Low	0	1
7f	146.17	1.38	-4.18	Moderately soluble	Low	0	1
7g	155.4	1.07	-4.47	Moderately soluble	Low	0	1

a- Topological polar surface area b- Log of partition coefficient (P), c- Log solubility, d-estimated aqueous solubility, e- Gastrointestinal.

**Table 2:** MICsby REMA plate method and IC50 for InhA inhibition

**Compound Code**	**Mtb MIC (μM)**	**InhA IC** **50 ** **(μM)**	**Compound Code**	**Mtb MIC (μM)**	**InhA IC** **50 ** **(μM)**
6a	12.5	0.68	6p	100	86
6b	**0.19**	**0.09**	6q	50	42
6c	3.12	6.15	6r	>100	>100
6d	25	10.78	6s	50	36
6e	12.5	5.95	6t	>100	>100
6f	12.5	1.2	6u	>100	>100
6g	**0.39**	**0.19**	6v	50	47
6h	6.25	0.34	7a	50	54
6i	**0.39**	**0.12**	7b	50	24
6j	6.25	3.1	7c	50	37
6k	12.5	6.36	7d	100	67
6l	50	23	7e	100	82
6m	12.5	6.3	7f	>100	98
6n	6.25	3.14	7g	>100	>100
6o	100	76	INH	0.05	NA

*2-(4-Amino-(3-pyridin-4-yl)-5-thioxo-4,5-dihydro-1H[1,2,4]triazol-1-yl)-N-(2-chloro-5-trifluoromethylphenyl) acetamide (6* ***k*** *)*

White amorphous solid; Recrystallizing solvent: Ethanol; Yield: 55%; M.p.: 184-186 °C; FT-IR (KBr, cm^-1^): 3344, 3261 (N-H stretch, Amine [NH_2_]), 3367 (N-H stretch, amide), 3047, 2989 (aromatic C-H stretch), 2929 (aliphatic C-H stretch, CH_2_), 1668 (C=O stretch, amide), 1334 (C-F stretch), 1180 (C=S stretch, triazole), 729 (C-Cl stretch); ^1^H NMR (600 MHz, DMSO-d_6_): δ 10.20 (s, 1H, NH), 8.72 (d, *J *= 4.8 Hz, 2H, pyridine), 8.25, (s, 1H, phenyl), 7.98 (d, *J *= 4.8 Hz, 2H, pyridine), 7.74 (d, *J *= 7.8 Hz, 1H, phenyl), 7.53 (d, *J *= 8.4 Hz, 1H, phenyl), 6.32 (s,2H, NH_2_), 4.27 (s, 2H, CH_2_); ^13^C NMR (100 MHz, DMSO-d_6_): δ 167.13 (1C, C-5 [C=S], triazole), 154.63 (1C, C=O, amide), 149.54 (1C, C-3, triazole), 152.03, 133.98, 121.27 (5C, pyridine), 135.46, 130.13, 128.59, 128.27, 124.67, 120.02 (6C, phenyl), 121.62 (1C, CF_3_), 35.72 (1C, CH_2_). Anal. Calc. for C_16_H_12_ClF_3_N_6_OS: C, 44.81; H, 2.82; N, 19.60.

*2-(4-Amino-(3-pyridin-4-yl)-5-thioxo-4,5-dihydro-1H[1,2,4]triazol-1-yl)-N-(3-methoxyphenyl) acetamide (6* ***l*** *)*

Yellow amorphous solid; Recrystallizing solvent: Isopropanol; Yield: 70%; M.p.: 222-224 ^o^C; FT-IR (KBr, cm^-1^): 3238, 3186 (N-H stretch, Amine [NH_2_]), 3331 (N-H stretch, amide), 3074, 3010 (aromatic C-H stretch), 2974, 2928, 2879 (aliphatic C-H stretch, CH_3_ and CH_2_), 1672 (C=O stretch, amide), 1282 (C-O-C stretch), 1201 (C=S stretch, triazole); ^1^H NMR (600 MHz, DMSO-d_6_): δ 10.32 (s, 1H, NH), 8.71 (d, *J *= 4.8 Hz, 2H, pyridine), 7.97 (d, *J *= 5.4 Hz, 2H, pyridine), 7.26 (s, 1H, phenyl), 7.20 (t, *J *= 8.1 Hz, 1H, phenyl), 7.09 (d, *J *= 8.4 Hz, 1H, phenyl), 6.63 (d, *J *= 7.8 Hz, 1H, phenyl), 6.31 (s,2H, NH_2_), 4.16 (s, 2H, CH_2_), 3.70 (s, 3H, CH_3_); ^13^C NMR (100 MHz, DMSO-d_6_): δ 165.79 (1C, C-5 [C=S], triazole), 154.41 (1C, C=O, amide), 149.49 (1C, C-3, triazole), 151.80, 134.03, 121.24 (5C, pyridine), 159.40, 139.56, 129.13, 111.45, 108.78, 105.11 (6C, phenyl), 54.72 (1C, CH_3_), 36.78 (1C, CH_2_). Anal. Calc. for C_16_H_16_N_6_O_2_S: C, 53.92; H, 4.52; N, 23.58.

*2-(4-Amino-(3-pyridin-4-yl)-5-thioxo-4,5-dihydro-1H[1,2,4]triazol-1-yl)-N-(4-fluorophenyl) acetamide (6* ***m*** *)*

Orange amorphous solid; Recrystallizing solvent: Ethanol; Yield: 72%; M.p.: 227-229 °C; FT-IR (KBr, cm^-1^): 3232, 3176 (N-H stretch, Amine [NH_2_]), 3290 (N-H stretch, amide), 3037 (aromatic C-H stretch), 2916, 2877 (aliphatic C-H stretch, CH_2_), 1660 (C=O stretch, amide), 1350 (C-F stretch), 1188 (C=S stretch, triazole); ^1^H NMR (600 MHz, DMSO-d_6_): δ 10.40 (s, 1H, NH), 8.71 (d, *J *= 4.8 Hz, 2H, pyridine), 7.97 (d, *J *= 4.8 Hz, 2H, pyridine), 7.57-7.59 (m, 2H, phenyl), 7.14 (t, *J *= 8.7 Hz, 2H, phenyl), 6.31 (s,2H, NH_2_), 4.16 (s, 2H, CH_2_); ^13^C NMR (100 MHz, DMSO-d_6_): δ 165.66 (1C, C-5 [C=S], triazole), 154.43 (1C, C=O, amide), 149.67 (1C, C-3, triazole), 151.84, 133.91, 121.20 (5C, pyridine), 156.98, 134.79, 120.89, 115.07 (6C, phenyl), 36.51 (1C, CH_2_). Anal. Calc. for C_15_H_13_FN_6_OS: C, 52.32; H, 3.81; N, 24.40.

*2-(4-Amino-(3-pyridin-4-yl)-5-thioxo-4,5-dihydro-1H[1,2,4]triazol-1-yl)-N-(4-trifluoromethylphenyl) acetamide (6* ***n*** *)*

Buff solid; Recrystallizing solvent: Ethanol; Yield: 90%; M.p.: 227-229 °C; FT-IR (KBr, cm^-1^): 3265, 3201 (N-H stretch, Amine [NH_2_]), 3362 (N-H stretch, amide), 3070, 3041 (aromatic C-H stretch), 2933(aliphatic C-H stretch, CH_2_), 1680 (C=O stretch, amide), 1317 (C-F stretch), 1172 (C=S stretch, triazole); ^1^H NMR (500 MHz, DMSO-d_6_): δ 10.74 (s, 1H, NH), 8.74 (d, *J *= 6.0Hz, 2H, pyridine), 8.00 (d, *J *= 6.0Hz, 2H, pyridine), 7.81 (d, *J *= 8.5 Hz, 2H, phenyl), 7.70 (d, *J *= 8.0Hz, 2H, phenyl), 6.35 (s,2H, NH_2_), 4.25 (s, 2H, CH_2_); ^13^C NMR (100 MHz, DMSO-d_6_): δ 166.33 (1C, C-5 [C=S], triazole), 154.57 (1C, C=O, amide), 149.28 (1C, C-3, triazole), 151.75, 133.88, 121.31 (5C, pyridine), 142.02, 134.33, 125.67, 118.90 (6C, phenyl), 124.33 (1C, CF_3_), 36.47 (1C, CH_2_). Anal. Calc. for C_16_H_13_F_3_N_6_OS: C, 48.73; H, 3.32; N, 21.31.

*2-(4-Amino-(3-pyridin-4-yl)-5-thioxo-4,5-dihydro-1H[1,2,4]triazol-1-yl)-N-pyridin-2-yl acetamide (6* ***o*** *)*

Pale yellow amorphous solid; Recrystallizing solvent: Isopropanol; Yield: 69%; M.p.: 212-214 °C; FT-IR (KBr, cm^-1^): 3261, 3171 (N-H stretch, Amine [NH_2_]), 3335 (N-H stretch, amide), 3105, 3063 (aromatic C-H stretch), 2980, 2933 (aliphatic C-H stretch, CH_2_), 1676 (C=O stretch, amide), 1188 (C=S stretch, triazole); ^1^H NMR (600 MHz, DMSO-d_6_): δ 10.77 (s, 1H, NH), 8.71 (d, *J *= 4.8 Hz, 2H, pyridine), 8.31 (d, *J *= 4.8 Hz, 1H, pyridine[amide]), 7.97 (d, *J *= 5.4 Hz, 2H, pyridine), 7.77 (t, *J *= 7.5 Hz, 1H, pyridine[amide]), 7.10 (t, *J *= 6.3 Hz, 2H, pyridine[amide]), 6.31 (s,2H, NH_2_), 4.22 (s, 2H, CH_2_); ^13^C NMR (100 MHz, DMSO-d_6_): δ 166.76 (1C, C-5 [C=S], triazole), 154.58 (1C, C=O, amide), 147.87 (1C, C-3, triazole), 149.95, 133.86, 121.24 (5C, pyridine), 151.94, 151.65, 138.08, 119.50, 113.42 (5C, pyridine [amide]), 36.83 (1C, CH_2_). Anal. Calc. for C_14_H_13_N_7_OS: C, 51.36; H, 4.00; N, 29.95.

*2-(4-Amino-(3-pyridin-4-yl)-5-thioxo-4,5-dihydro-1H[1,2,4]triazol-1-yl)-N-(6-methylpyridin-2-yl) acetamide (6* ***p*** *)*

Dark brown amorphous solid; Recrystallizing solvent: Isopropanol; Yield: 56%; M.p.: 230-232 °C; FT-IR (KBr, cm^-1^): 3271, 3228 (N-H stretch, Amine [NH_2_]), 3346 (N-H stretch, amide), 3140, 3047 (aromatic C-H stretch), 2962, 2920, 2862 (aliphatic C-H stretch, CH_3_ and CH_2_), 1680 (C=O stretch, amide), 1190 (C=S stretch, triazole); ^1^H NMR (600 MHz, DMSO-d_6_): δ 10.71 (s, 1H, NH), 8.71 (d, *J *= 4.8 Hz, 2H, pyridine), 7.97 (d, *J *= 5.4 Hz, 2H, pyridine), 7.83 (d, *J *= 7.2 Hz, 1H, pyridine[amide]), 7.64 (t, *J *= 8.1 Hz, 1H, pyridine[amide]), 6.97 (d, *J *= 7.2 Hz, 1H, pyridine[amide]), 6.30 (s,2H, NH_2_), 4.19 (s, 2H, CH_2_), 2.39 (s, 3H, CH_3_); ^13^C NMR (100 MHz, DMSO-d_6_): δ 166.57 (1C, C-5 [C=S], triazole), 154.29 (1C, C=O, amide), 149.77 (1C, C-3, triazole), 150.90, 133.89, 121.19 (5C, pyridine), 156.32, 151.84, 138.13, 118.63, 110.34 (5C, pyridine [amide]), 36.34 (1C, CH_2_), 23.53 (1C, CH_3_). Anal. Calc. for C_15_H_15_N_7_OS: C, 52.77; H, 4.43; N, 28.72.

*2-(4-Amino-(3-pyridin-4-yl)-5-thioxo-4,5-dihydro-1H[1,2,4]triazol-1-yl)-N-(4-methylpyridin-2-yl) acetamide (6* ***q*** *)*

Buff solid; Recrystallizing solvent: Ethanol; Yield: 63%; M.p.: 217-219 °C; FT-IR (KBr, cm^-1^): 3271, 3236 (N-H stretch, Amine [NH_2_]), 3340 (N-H stretch, amide), 3194, 3146 (aromatic C-H stretch), 2976, 2929, 2847 (aliphatic C-H stretch, CH_3_ and CH_2_), 1680 (C=O stretch, amide), 1193 (C=S stretch, triazole); ^1^H NMR (500 MHz, DMSO-d_6_): δ 10.71 (s, 1H, NH), 8.74 (d, *J *= 6.0Hz, 2H, pyridine), 8.19 (d, *J *= 5.0 Hz, 1H, pyridine[amide]), 8.01 (d, *J *= 6.0Hz, 2H, pyridine), 7.90 (s, 1H, pyridine[amide]), 6.97 (d, *J *= 5.0Hz, 1H, pyridine[amide]), 6.34 (s,2H, NH_2_), 4.24 (s, 2H, CH_2_), 2.31 (s, 3H, CH_3_); ^13^C NMR (100 MHz, DMSO-d_6_): δ 166.61 (1C, C-5 [C=S], triazole), 154.47 (1C, C=O, amide), 149.87 (1C, C-3, triazole), 151.88, 133.88, 121.20 (5C, pyridine), 151.69, 148.72, 147.39, 120.44, 113.83 (5C, pyridine [amide]), 36.19 (1C, CH_2_), 20.90 (1C, CH_3_). Anal. Calc. for C_15_H_15_N_7_OS: C, 52.77; H, 4.43; N, 28.72.

*2-(4-Amino-(3-pyridin-4-yl)-5-thioxo-4,5-dihydro-1H[1,2,4]triazol-1-yl)-N-pyrimidin-2-yl acetamide (6* ***r*** *)*

Brown amorphous solid; Recrystallizing solvent: Ethanol; Yield: 45%; M.p.: 205-207 °C; FT-IR (KBr, cm^-1^): 3286, 3203 (N-H stretch, Amine [NH_2_]), 3356 (N-H stretch, amide), 3091, 3053 (aromatic C-H stretch), 2974, 2924 (aliphatic C-H stretch, CH_2_), 1670 (C=O stretch, amide), 1190 (C=S stretch, triazole); ^1^H NMR (500 MHz, DMSO-d_6_): δ 10.95 (s, 1H, NH), 8.74 (d, *J *= 5.5 Hz, 2H, pyridine), 8.68 (d, *J *= 4.5 Hz, 2H, pyrimidine), 8.00 (d, *J *= 5.5 Hz, 2H, pyridine), 7.21 (t, *J *= 4.5 Hz, 1H, pyrimidine), 6.33 (s,2H, NH_2_), 4.42 (s, 2H, CH_2_); ^13^C NMR (100 MHz, DMSO-d_6_): δ 163.42 (1C, C-5 [C=S], triazole), 154.45 (1C, C=O, amide), 149.21 (1C, C-3, triazole), 151.64, 134.63, 121.30 (5C, pyridine), 157.96, 157.40, 116.41 (4C, pyrimidine), 37.93 (1C, CH_2_). Anal. Calc. for C_13_H_12_N_8_OS: C, 47.55; H, 3.68; N, 34.13.

*2-(4-Amino-(3-pyridin-4-yl)-5-thioxo-4,5-dihydro-1H[1,2,4]triazol-1-yl)-N-(5-methylisoxazol-3-yl) acetamide (6* ***s*** *)*

Pale yellow amorphous solid; Recrystallizing solvent: Isopropanol; Yield: 56%; M.p.: 242-244 °C; FT-IR (KBr, cm^-1^): 3236, 3136 (N-H stretch, Amine [NH_2_]), 3342 (N-H stretch, amide), 3056, 2953, 2918 (aliphatic C-H stretch, CH_3 _and CH_2_), 1678 (C=O stretch, amide), 1570 (aromatic C=N stretch) 1199 (C=S stretch, triazole); ^1^H NMR (600 MHz, DMSO-d_6_): δ 11.27 (s, 1H, NH), 8.74 (d, *J *= 5.4 Hz, 2H, pyridine), 7.99 (d, *J *= 5.4 Hz, 2H, pyridine), 6.61 (s, 1H, isoxazole), 6.33 (s, 2H, NH_2_), 4.20 (s, 2H, CH_2_), 2.38 (s, 3H, CH_3_); ^13^C NMR (100 MHz, DMSO-d_6_): δ 166.19 (1C, C-5 [C=S], triazole), 154.44 (1C, C=O, amide), 149.93 (1C, C-3, triazole), 149.66, 133.86, 121.21 (5C, pyridine), 169.34, 157.85, 96.13 (3C, isoxazole), 35.44 (1C, CH_2_), 12.18 (1C, CH_3_). Anal. Calc. for C_13_H_13_N_7_O_2_S: C, 47.12; H, 3.95; N, 29.59.

*2-(4-Amino-(3-pyridin-4-yl)-5-thioxo-4,5-dihydro-1H[1,2,4]triazol-1-yl)-N-(4-methylbenzothiazol-3-yl) acetamide (6* ***t*** *)*

White amorphous solid; Recrystallizing solvent: Isopropanol; Yield: 52%; M.p.: 243-245 °C; FT-IR (KBr, cm^-1^): 3261, 3184 (N-H stretch, Amine [NH_2_]), 3331 (N-H stretch, amide), 3109, 3049 (aromatic C-H stretch), 2953, 2916, 2818 (aliphatic C-H stretch, CH_3 _and CH_2_), 1685 (C=O stretch, amide), 1188 (C=S stretch, triazole); ^1^H NMR (600 MHz, DMSO-d_6_): δ12.76 (s, 1H, NH), 8.71 (d, *J *= 5.4 Hz, 2H, pyridine), 7.96 (d, *J *= 5.4 Hz, 2H, pyridine), 7.76 (d, *J *= 7.8 Hz, 1H, benzothiazole), 7.25 (d, *J *= 7.2 Hz, 1H, benzothiazole), 7.19 (t, *J *= 7.5 Hz, 1H, benzothiazole), 6.33 (s, 2H, NH_2_), 4.31 (s, 2H, CH_2_), 3.29 (s, 3H, CH_3_); ^13^C NMR (100 MHz, DMSO-d_6_): δ 167.12 (1C, C-5 [C=S], triazole), 154.37 (1C, C=O, amide), 147.58 (1C, C-3, triazole), 149.95, 133.83, 121.22 (5C, pyridine), 159.79, 152.06, 131.10, 129.88, 126.50, 123.45, 118.87 (7C, benzothiazole), 34.73 (1C, CH_2_), 25.38 (1C, CH_3_). Anal. Calc. for C_17_H_15_N_7_OS_2_: C, 51.37; H, 3.80; N, 24.67.

*2-(4-Amino-(3-pyridin-4-yl)-5-thioxo-4,5-dihydro-1H[1,2,4]triazol-1-yl)-N-thiazol-2-yl acetamide (6* ***u*** *)*

Orange amorphous solid; Recrystallizing solvent: Ethanol; Yield: 42%; M.p.: 253-255^o^C; FT-IR (KBr, cm^-1^): 3327, 3284 (N-H stretch, Amine [NH_2_]), 3342 (N-H stretch, amide), 3151, 3099 (aromatic and heteroaromatic C-H stretch), 3051 (aliphatic C-H stretch, CH_2_), 1683 (C=O stretch, amide), 1174 (C=S stretch, triazole); ^1^H NMR (600 MHz, DMSO-d_6_): δ 12.42 (s, 1H, NH), 8.71 (d, *J *= 5.4 Hz, 2H, pyridine), 7.96 (d, *J *= 5.4 Hz, 2H, pyridine), 7.47 (d, *J *= 3.0Hz, 1H, thiazole), 7.22 (d, *J *= 3.6 Hz, 1H, thiazole), 6.32 (s, 2H, NH_2_), 4.26 (s, 2H, CH_2_); ^13^C NMR (100 MHz, DMSO-d_6_): δ 166.10 (1C, C-5 [C=S], triazole), 154.42 (1C, C=O, amide), 149.93 (1C, C-3, triazole), 152.03, 133.83, 121.22 (5C, pyridine), 157.79, 137.57, 113.45 (3C, thiazole), 34.60 (1C, CH_2_). Anal. Calc. for C_12_H_11_N_7_OS_2_: C, 43.23; H, 3.33; N, 29.41.

*2-(4-Amino-(3-pyridin-4-yl)-5-thioxo-4,5-dihydro-1H[1,2,4]triazol-1-yl)-N-(5-methylthiazol-2-yl) acetamide (6* ***v*** *)*

Yellow amorphous solid; Recrystallizing solvent: Ethanol; Yield: 63%; M.p.: 250-252^o^C; FT-IR (KBr, cm^-1^): 3281, 3192 (N-H stretch, Amine [NH_2_]), 3360 (N-H stretch, amide), 3037 (aromatic C-H stretch), 2956, 2920 (aliphatic C-H stretch, CH_3 _and CH_2_), 1687 (C=O stretch, amide), 1178 (C=S stretch, triazole); ^1^H NMR (600 MHz, DMSO-d_6_): δ 12.21 (s, 1H, NH), 8.71 (d, *J *= 5.4 Hz, 2H, pyridine), 7.96 (d, *J *= 5.4 Hz, 2H, pyridine), 7.13 (s, 1H, thiazole), 6.31 (s, 2H, NH_2_), 4.23 (s, 2H, CH_2_), 2.32 (s, 3H, CH_3_); ^13^C NMR (100 MHz, DMSO-d_6_): δ 165.80 (1C, C-5 [C=S], triazole), 154.44 (1C, C=O, amide), 149.97 (1C, C-3, triazole), 152.02, 133.83, 121.22 (5C, pyridine), 155.96, 134.73, 126.33 (3C, thiazole), 35.54 (1C, CH_2_), 11.09 (1C, CH_3_). Anal. Calc. for C_13_H_13_N_7_OS_2_: C, 44.94; H, 3.77; N, 28.22.

*General synthetic procedure for the preparation of* ***7a-7g.***

A mixture of 5-pyridin-4-yl-3H-(1,3,4)-oxadiazole-2-thione (**4**)(0.05 mol) and 2-chloro-N-(aryl or heteroaryl) acetamides (**2o, 2s, 2u-2y**) (0.05 mol) along with anhydrous potassium carbonate (K_2_CO_3_) (0.075 mol) was stirred in dimethyl formamide (DMF) at room temperature. After completion of reaction (monitored by TLC), ice cold water was added to precipitate out the product which was collected and purified by recrystallization from ethanol.

*2-(5-pyridin-4-yl-2-thioxo-[1,3,4]oxadiazol-3-yl)-N-pyridin-2-yl acetamide (* ***7a*** *)*

Brown crystalline solid; Yield: 45%; M.p.: 216-218^o^C; FT-IR (KBr, cm^-1^): 3204 (N-H stretch, amide), 3161, 3113 (aromatic C-H stretch), 2980, 2935 (aliphatic C-H stretch, CH_2_), 1682 (C=O stretch, amide), 1180 (C=S stretch, oxadiazole); ^1^H NMR (300 MHz, DMSO-d_6_): δ 10.94 (s, 1H, NH), 8.81 (dd, *J *= 4.5, 3.0Hz, 2H, pyridine), 8.34 (d, *J *= 4.8 Hz, 1H, pyridine [amide]), 8.02 (d, *J *= 8.4 Hz, 1H, pyridine [amide]), 7.88 (dd, *J *= 4.2, 2.7 Hz, 2H, pyridine), 7.76-7.82 (m, 1H, pyridine [amide]), 7.11-7.15 (m,1H, pyridine [amide]), 4.43 (s, 2H, CH_2_); ^13^C NMR (75 MHz, DMSO-d_6_): δ165.79 (1C, C=O, amide), 164.78 (1C, C-2 [C=S], oxadiazole), 163.65 (1C, C-5, oxadiazole), 148.11, 129.99, 119.94 (5C, pyridine), 151.54, 150.91, 138.38, 119.83, 113.47 (5C, pyridine [amide]), 36.61 (1C, CH_2_). Anal. Calc. for C_14_H_11_N_5_O_2_S: C, 53.66; H, 3.54; N, 22.35.

*N-(5-methylisoxazol-3-yl)-2-(5-(pyridin-4-yl)-2-thioxo-1,3,4-oxadiazol-3(2H)-yl)acetamide (* ***7b*** *)*

White crystalline solid; Yield: 68%; M.p.: 232-234 °C; FT-IR (KBr, cm^-1^): 3263 (N-H stretch, amide), 3018, 2972, 2912 (aliphatic C-H stretch, CH_3 _and CH_2_), 1691 (C=O stretch, amide), 1572 (aromatic C=N stretch) 1172 (C=S stretch, oxadiazole); ^1^H NMR (400 MHz, DMSO-d_6_): δ 11.42 (s, 1H, NH), 8.82 (d, *J *= 8Hz, 2H, pyridine), 7.89 (d, *J *= 4Hz, 2H, pyridine), 6.61 (s, 1H, isoxazole), 4.39 (s, 2H, CH_2_), 2.38 (s, 3H, CH_3_); ^13^C NMR (75 MHz, DMSO-d_6_): δ165.28 (1C, C=O, amide), 164.67 (1C, C-2 [C=S], oxadiazole), 163.69 (1C, C-5, oxadiazole), 150.94, 129.99, 119.95 (5C, pyridine), 169.89, 157.82, 96.17 (3C, isoxazole), 36.13 (1C, CH_2_), 12.11 (1C, CH_3_). Anal. Calc. forC_13_H_11_N_5_O_3_S: C, 49.21; H, 3.49; N, 22.07.

*2-(5-pyridin-4-yl-2-thioxo-[1,3,4]oxadiazol-3-yl)-N-thiazol-2-yl acetamide (7* ***c*** *)*

White crystalline solid; Yield: 61%; M.p.: 244-246 °C; FT-IR (KBr, cm^-1^): 3194 (N-H stretch, amide), 3053 (aromatic C-H stretch), 2949, 2920 (aliphatic C-H stretch, CH_2_), 1695 (C=O stretch, amide), 1174 (C=S stretch, oxadiazole); ^1^H NMR (300 MHz, DMSO-d_6_): δ11.41 (s, 1H, NH), 8.80 (dd, *J *= 4.2, 2.7Hz, 2H, pyridine), 7.87 (dd, *J *= 4.5, 3Hz, 2H, pyridine), 7.50 (d, *J *= 3.6 Hz, 1H, thiazole), 7.25 (d, *J *= 3.3Hz, 1H, thiazole), 4.44 (s, 2H, CH_2_); ^13^C NMR (75 MHz, DMSO-d_6_): δ165.25 (1C, C=O, amide), 164.54 (1C, C-2 [C=S], oxadiaazole), 163.76 (1C, C-5, oxadiazole), 150.93, 129.98, 119.96 (5C, pyridine), 157.70, 137.79, 113.95 (3C, thiazole), 35.42 (1C, CH_2_). Anal. Calc. for C_12_H_9_N_5_O_2_S_2_: C, 45.13; H, 2.84; N, 21.93.

*N-(5-methylthiazol-2-yl)-2-(5-(pyridin-4-yl)-2-thioxo-1,3,4-oxadiazol-3(2H)-yl)acetamide (* ***7d*** *)*

White crystalline solid; Yield: 47%; M.p.: 236-238 °C; FT-IR (KBr, cm^-1^): 3371 (N-H stretch, amide), 3184, 3061 (aromatic C-H stretch), 2820 (aliphatic C-H stretch, CH_3 _and CH_2_), 1691 (C=O stretch, amide), 1168 (C=S stretch, oxadiazole); ^1^H NMR (300 MHz, DMSO-d_6_): δ 11.20 (s, 1H, NH), 8.81 (d, *J *= 5.4 Hz, 2H, pyridine), 7.88 (d, *J *= 5.4 Hz, 2H, pyridine), 7.17 (s, 1H, thiazole), 4.43 (s, 2H, CH_2_), 2.35 (s, 3H, CH_3_); ^13^C NMR (75 MHz, DMSO-d_6_): δ164.93 (1C, C=O, amide), 164.54 (1C, C-2 [C=S], oxadiazole), 163.74 (1C, C-5, oxadiazole), 150.92, 129.97, 119.95 (5C, pyridine), 155.96, 134.67, 126.71 (3C, thiazole), 35.41 (1C, CH_2_), 11.09 (1C, CH_3_). Anal. Calc. for C_13_H_11_N_5_O_2_S_2_: C, 46.83; H, 3.33; N, 21.01.

*N-(5-nitrothiazol-2-yl)-2-(5-(pyridin-4-yl)-2-thioxo-1,3,4-oxadiazol-3(2H)-yl)acetamide (* ***7e*** *)*

Brown crystalline solid; Yield: 56%; M.p: 254-256 ^o^C; FT-IR (KBr, cm^-1^): 3250 (N-H stretch, amide), 3076 (aromatic C-H stretch), 2933 (aliphatic C-H stretch, CH_2_), 1635 (C=O stretch, amide), 1161 (C=S stretch, oxadiazole); ^1^H NMR (400 MHz, DMSO-d_6_): δ13.57 (s, 1H, NH), 8.82 (d, *J *= 4Hz, 2H, pyridine), 8.68 (s, 1H, thiazole), 7.89 (d, *J *=4 Hz, 2H, pyridine), 4.54 (s, 2H, CH_2_); ^13^C NMR (75 MHz, DMSO-d_6_): δ167.36 (1C, C=O, amide), 164.35 (1C, C-2 [C=S], oxadiazole), 163.88 (1C, C-5, oxadiazole), 150.95, 129.98, 119.99 (5C, pyridine), 161.36,142.67, 142.17 (3C, thiazole), 38.88 (1C, CH_2_). Anal. Calc. for C_12_H_8_N_6_O_4_S_2_: C, 39.56; H, 2.21; N, 23.07.

*2-(5-pyridin-4-yl-2-thioxo-[1,3,4]oxadiazol-3-yl)-N-benzothiazol-2-yl acetamide (* ***7f*** *)*

Yellow crystalline solid; Yield: 53%; M.P.: 206-208 °C; FT-IR (KBr, cm^-1^): 3184 (N-H stretch, amide), 3083 (aromatic C-H stretch), 2921 (aliphatic C-H stretch, CH_2_), 1689 (C=O stretch, amide), 1167 (C=S stretch, oxadiazole); ^1^H NMR (300 MHz, DMSO-d_6_): δ10.23 (s, 1H, NH),8.81 (d, *J *= 2.4 Hz, 2H, benzothiazole), 8.79 (dd, *J *= 4.65, 3.0 Hz, 2H, pyridine), 8.18 (dd,*J *= 9.0, 6.6 Hz, 1H, benzothiazole), 7.90 (dd, *J *= 4.2, 2.7 Hz, 2H, pyridine), 7.71 (d, *J *= 9.0 Hz, 1H, benzothiazole), 4.41 (s, 2H, CH_2_); ^13^C NMR (75 MHz, DMSO-d_6_): δ 171.72 (1C, C=O, amide), 164.35 (1C, C-2 [C=S], oxadiazole), 163.30 (1C, C-5, oxadiazole), 150.90, 130.15, 119.95 (5C, pyridine), 140.76, 132.98, 120.88, 118.28, 117.58 (7C, benzothiazole), 38.66 (1C, CH_2_). Anal. Calc. for C_16_H_11_N_5_O_2_S_2_: C, 52.02; H, 3.00; N, 18.96.

*N-(6-ethoxybenzo[d]thiazol-2-yl)-2-(5-(pyridin-4-yl)-2-thioxo-1,3,4-oxadiazol-3(2H)-yl)acetamide (* ***7g*** *)*

White crystalline solid; Yield: 56%; M.p.: 252-254 °C; FT-IR (KBr, cm^-1^): 3192 (N-H stretch, amide), 3078, 3052 (aromatic C-H stretch), 2747 (aliphatic C-H stretch, CH_2_), 1696 (C=O stretch, amide), 1177 (C=S stretch, oxadiazole); ^1^H NMR (300 MHz, DMSO-d_6_): δ10.67 (s, 1H, NH), 8.78 (dd, *J *= 4.5, 3.0 Hz, 2H, pyridine), 7.88 (dd, *J *= 4.5, 2.7 Hz, 2H, pyridine), 7.66 (d, *J *= 9.0 Hz, 1H, benzothiazole), 7.54 (d, *J *= 2.7 Hz 1H, benzothiazole), 7.03 (dd, *J *= 9.0, 6.3 Hz 1H, benzothiazole), 4.48 (s, 2H, CH_2_), 4.05 (q, 2H, OCH_2_), 1.33 (s, 3H, CH_3_); ^13^C NMR (75 MHz, DMSO-d_6_): δ 166.20 (1C, C=O, amide), 164.30 (1C, C-2 [C=S], oxadiazole), 163.76 (1C, C-5, oxadiazole), 150.92, 129.99, 119.97 (5C, pyridine), 155.40, 132.79, 120.21, 121.20, 115.33, 105.35 (7C, benzothiazole), 63.57 (1C, O-CH_2_), 35.86 (1C, CH_2_), 14.68 (1C, CH_3_). Anal. Calc. for C_18_H_15_N_5_O_3_S_2_: C, 52.29; H, 3.66; N, 16.94.

*Biological evaluation*

*Mtb H*_37_ *Rv assay [Resazurin microtiter-based assay (REMA)]*

To determine the potency of a compound against *M. tuberculosis*, the compounds were dissolved and serially diluted in DMSO in a 384 well plate. 1 μL of compound was dispensed per well for a 10-point concentration response format using a BiomekFx liquid dispenser. To this, 40 μL of *M. tuberculosis *(3-5 x 105 CFU/mL in 7H9 medium, 0.05% Tween 80, ADC, Casamino acids) were added with a multidrop dispenser. The plates were then incubated at 37 °C for 6 days. 10 μL of resazurin solution (20 mg/100 mL diluted 1:1 with 10% Tween 80) were added and the plates were incubated for an additional 24 h at 37 °C for color development. Absorbance in Spectramax at two wavelengths (575 & 610 nm) was measured and the MIC determined (Ratio of the absorbance values used for calculating 90% inhibition with respect to growth control).

*Mtb InhA enzyme inhibition assay (Fluorescence based assay)*

The compounds were dissolved in 100% dimethyl sulfoxide (DMSO). InhA (1.25 nM) was preincubated for ~15 min at room temperature with 0.050 mM NADH and inhibitor at a final concentration of 2% (v/v) dimethyl sulfoxide in 50μL reaction volume. The 50μL enzyme reaction contained PIPES- 30 mM, pH 7.5, NaCl - 50 mM, 0.005% Brij-35, DTT - 2mM, and EDTA- 0.1mM. The enzyme reaction was started by the addition of 150μl of dodecyl CoA synthesized in-house and the depletion of NADH was followed by measuring the fluorescence of NADH kinetically in the reaction for 30min at excitation at 340nm and emission at 420nm using Tecan Saffire II reader. The concentration at which 50% inhibition in enzyme activity was observed was reported as the IC_50_.

## Results and Discussion

*Designing*

Pan *et al*. elucidated a general binding model based on the binding modes of known InhA inhibitors and identified three key areas of interaction at the InhA active site (10). The first area includes the groups involved in the hydrogen bond interactions amongst the groups present on the inhibitors, *i.e.,* Tyr-158 and the 2’-hydroxyl functionality of nicotinamide ribose. The second region is the hydrophobic pocket wherein the hydrophobic contacts include Gly-96, Phe-97, Met-103, Gly-104, Phe-149, Ala-157, Met-161, Pro-193, Ala-198, Met-199, Ile-202, Val-203, Ile215, and Leu218 residues. The third region is present near the hydrogen bonding group of the inhibitor. This region is comparatively size constrained and exposed to solvent. It is also closer to non-polar as well as polar groups (cofactor phosphodiester bridge), in the substrate binding loop (eg. Ala-198). The selectivity of enzyme inhibition is extremely sensitive to the size and chemical nature of the substituent.

One important class among the previously identified anti-mycobacterial hits are the pyrrolidine carboxamide class of direct InhA inhibitors. Their overall chemical structure topology incorporates ring C as the hydrophobic binding region, a linker extending to six atom length, a hydrogen bonding carbonyl incorporated into ring B and finally, ring A serving as the size constrained region (Figure 2).

In the present methodology, we hybridized in one molecular platform, the 1,2,4-triazole-5-thione core/linker to hydrogen bonding carbonyl group of amides to give the designed compound **6**. The designed ligand thus has three parts:1,2,4-triazole-5-thione moiety (ring B) linked with acetamido group sandwiched between a left-hand pyridine (ring C) and a right-hand substituted aryl/heteroaryl moiety (ring A) as shown in Figure 2. The presence of a pyridine nucleus in INH as hydrophobic binding region in INH-NAD^+^ adduct made it an automatic choice as the left-hand ring A (6) The selection of 1,2,4-triazole-5-thione nucleus as ring B was based on the established literature wherein 1,2,4-triazole nucleus has been proved to elicit anti-mycobacterial activity (20, 21). The selection of the right-hand substituent was purely based on the inputs from the review by Pan *et al.*, wherein it was found that the TB activity could be optimized at the right-hand side.

*SwissADME predictions*

Drug development consists of calculation of ADME profile, but determination of ADME by actual experimentations for all the compounds is very time consuming and tedious task. In this situation, computer-models offer valid replacements to (22) Thus, SwissADME web tool is freely available and gives access to the fast and robust models for prediction of pharmacokinetics/physicochemical properties and drug-likeness. Easy efficient input and interpretation are key advantages of the tool. We have used this tool to predict properties of our compounds. All the compounds were observed to follow the Lipinski’s rule of five (Table 1). Judging the data, the molecules seem to be drug like and may have good passive oral absorption.

An intuitive method offered by SwissADME model is known as BOILED-Egg, which simultaneously predicts two crucial ADME parameters, *viz*. brain access (BBB) and passive gastrointestinal absorption (HIA). Although conceptually it looks very simple, as it relies only on two descriptors, WLOGP (lipophilicity) and TPSA (apparent polarity), the BOILED-Egg has been verified to be a candid explanation and competent translation to molecular design in numerous drug discoveries (23). Out of 29 compounds, none was observed in egg yolk (Yellow) region, predicting that all do not penetrate the brain. 19 compounds were observed to be absorbed well but not crossing BBB (in the white), in all these molecules Ar/Het substitution was phenyl/substituted phenyl moiety. 

Remaining 10molecules (8r- 8v, 13c-13g) were observed to be not absorbed and not crossing BBB (outside the Egg) and may have poor bioavailability (Figure 3). When we closely observed the Ar/Het substitution, we found that all these 10 molecules have substituted/unsubstituted five/six membered heterocycle or substituted/unsubstituted heteroaryl fused ring system such as benzothiazole. Moreover, in biological screening these 10 molecules were found inactive or nearly inactive in both Mtb and InhA enzyme inhibition assay.

*Chemistry*

The synthetic pathway used to achieve the target compounds has been delineated in Scheme 1. The construction of the target compounds, **6a-6v**, involved the synthesis of 5-(pyridin-4-yl)-3H-(1,3,4)-oxadiazole-2-thione (**4**) by refluxing isoniazid with carbon disulfide in the presence of ethanolic KOH. Compound **4** on further treatment with 99% hydrazine hydrate yielded 4-amino-3-(pyridin-4-yl)-1H-(1,2,4)-triazole-5-thione (**5**). Both **4** and **5** were obtained in good yield. It has been observed that compound **4** can undergo thiol-thione tautomerism and thereby could also exist in thiol form (24). However, compounds **4 **and **5 **exist in thione form which was confirmed by FT-IR and ^13^C-NMR data. Compounds **2a-2y** were obtained by N-chloroacetylation of various aromatic and heteroaromatic amines (**1a-1y**) using weak base such as K_2_CO_3 _in dichloromethane (DCM) as solvent. The final step involved the condensation between **2a-2v** and **5** mediated by anhydrous K_2_CO_3_ and DMF to afford the target compounds **6a-6v**. Compounds** 7a-7g** from condensation between **4 **and **2o, 2s, 2u-2y **were also attempted in synthesis, as steps towards the derivation of structure-activity relationships (SAR) and lead identification. The reactions were monitored for completion by thin layer chromatography. The structures of newly synthesized compounds were confirmed by spectral data -^1^H NMR,^13^C NMR, and FTIR.

FT-IR spectra of these derivatives displayed characteristic absorption in the range of 3360-3176 cm^-1^ corresponding to NH_(s)_ vibration of free amino group as well as NH_(s)_ vibration of the amide group. In addition, they also exhibited characteristic absorption peak in the range of 1687-1660 cm^-1^ corresponding to C=O_(s)_ of amide group and in the range of 1213-1172 cm^-1^ corresponding to C=S_(s)_. Proton NMR spectra of compounds **6a-6v **showed two characteristic doublets in a region of δ=8.71-8.74 and 7.96-8.01 ppm corresponding to four hydrogen atoms of the pyridine ring system and three characteristic singlets in the region of δ=6.31-6.34, 4.16-4.27, and 10.20-12.42 ppm corresponding to protons of the free NH_2_ group, linker -CH_2_- group, and NH group of amide linkage respectively. The spectra also showed various peaks in the aromatic and aliphatic regions corresponding to the protons of variously substituted aromatic and heteroaromatic groups attached to the N atom of amide linkage. Furthermore, ^13^C NMR spectra of compounds **6a-6v **elicited characteristic peaks of C=S at δ=165-167 ppm, C=O at δ=154-155 ppm, C=N at δ=149-150 ppm, and of linker -CH_2_- at δ=34-37 ppm. They also exhibited three peaks at δ=151-152, 133-134, and 121 ppm corresponding to five carbon atoms of the pyridine ring and various other peaks corresponding to carbon atoms of aromatic or heteroaromatic substitution at the N atom of amide linkage. The characterization data of oxadiazole-based compounds **7a-7g** were interpreted in a similar fashion as that of their triazole counterparts.

*Biological evaluation*

The MIC and IC_50 _values have been shown in Table 2. From the data it was observed that there is direct correlation between the antitubercular activity and InhA inhibition. The molecules which exhibited higher InhA inhibition have also led to higher antitubercular activity in Mtb assay. Similarly compounds with less inhibition of InhA have shown poor antitubercular activity. This indicates that the antitubercular activity is due to the inhibition of InhA enzyme and thus we can say that our newly designed compounds specifically target InhA enzyme (Figure. 4)

In the aromatic series of 1,2,4-triazole-5-thiones, compounds **6b,** and **6g-6i** showed the best activity both in terms of MIC and InhA IC_50_ while the activity decreased in compounds **6c-6f** and **6j-6n.** The plausible reason could be justified by considering compound **6a **as the reference. The unsubstituted phenyl ring of **6a** does not show any remarkable inductive effect whereas the electron donating methyl group present at 4-position and 3,4-positions of the compounds **6b **and **6g** respectively elicits positive inductive (+I) effect which seems to be essential for the activation of the carbonyl group due to which it would form more stable hydrogen bond network with Tyr158 and NADH residues. In case of compounds **6c-6f** and **6j-6n** the presence of electron withdrawing groups on phenyl ring leads to negative inductive (-I) effect which seems to be unfavorable for activity as the carbonyl group would not be that much activated and the same is reflected in a mild decrease in the activity.

However, moving on from six-membered aromatic to six-membered and five-membered heterocyclic ring systems (**6o-6v**), the activity drastically decreases with compound **6t **being inactive. The inactivity of **6t **can be attributed to two reasons; the first being the strong electron withdrawing thiazole ring of benzothiazole and the second reason could be the bulk of the overall benzothiazole ring. 

Considering the seven oxadiazole molecules **7a-7g**, compounds** 7a-7e **are very slightly active while **7f-7g **are inactive thereby indicating that there is no significant change in the activity when compared to their triazole counterparts. This suggests that the size of ring A has more dominating effect on the final activity compared to the type of linker viz, triazole or oxadiazole.

As discussed in the designing section, the ring A is the size constrained region and therefore any increase or decrease in the size of the ring which form the optimum, would adversely affect the final activity of the compound. Thus, based on the results obtained, the optimum size of the ring A seems to be six-membered aromatic ring.

## Conclusions

Various 1,2,4-triazole derivatives targeting InhA were designed, synthesized and spectrally characterized using IR, ^1^H NMR and ^13^C NMR spectroscopy. The resazurin microtiter assay (REMA) of the characterized compounds on the *Mycobacterium tuberculosis *H_37_Rv strain identified promising candidates in the series and their mechanism of action validated by the InhA enzyme inhibitory studies. The compounds **6b, 6g-6i** were found to be promising and compound **6b** was identified as hit with Mtb H_37_Rv MIC of 0.19 µM and InhA IC_50_ value of 0.09 µM (90 nM). SwissADME predictions were found to be correlating structures with the biological activity and most of the compounds exhibited drug-likeliness.

There is a scope for further optimization of the identified hits to obtain more potent direct InhA inhibitors, which could serve as the plausible leads for further drug development.
